# Cannabinoids, reward processing, and psychosis

**DOI:** 10.1007/s00213-021-05801-2

**Published:** 2021-03-01

**Authors:** Brandon Gunasekera, Kelly Diederen, Sagnik Bhattacharyya

**Affiliations:** Department of Psychosis Studies, Institute of Psychiatry, Psychology and Neuroscience, King’s College London, 16 De Crespigny Park, Box P067, London, SE5 8AF UK

**Keywords:** Cannabis, Cannabidiol, CBD, THC, Dopamine, PET, fMRI, Psychosis, Schizophrenia, Reward processing, Aberrant salience

## Abstract

**Background:**

Evidence suggests that an overlap exists between the neurobiology of psychotic disorders and the effects of cannabinoids on neurocognitive and neurochemical substrates involved in reward processing.

**Aims:**

We investigate whether the psychotomimetic effects of delta-9-tetrahydrocannabinol (THC) and the antipsychotic potential of cannabidiol (CBD) are underpinned by their effects on the reward system and dopamine.

**Methods:**

This narrative review focuses on the overlap between altered dopamine signalling and reward processing induced by cannabinoids, pre-clinically and in humans. A systematic search was conducted of acute cannabinoid drug-challenge studies using neuroimaging in healthy subjects and those with psychosis

**Results:**

There is evidence of increased striatal presynaptic dopamine synthesis and release in psychosis, as well as abnormal engagement of the striatum during reward processing. Although, acute THC challenges have elicited a modest effect on striatal dopamine, cannabis users generally indicate impaired presynaptic dopaminergic function. Functional MRI studies have identified that a single dose of THC may modulate regions involved in reward and salience processing such as the striatum, midbrain, insular, and anterior cingulate, with some effects correlating with the severity of THC-induced psychotic symptoms. CBD may modulate brain regions involved in reward/salience processing in an opposite direction to that of THC.

**Conclusions:**

There is evidence to suggest modulation of reward processing and its neural substrates by THC and CBD. Whether such effects underlie the psychotomimetic/antipsychotic effects of these cannabinoids remains unclear. Future research should address these unanswered questions to understand the relationship between endocannabinoid dysfunction, reward processing abnormalities, and psychosis.

## Introduction

Since the nineteenth century description by Moreau of the psychotomimetic effects of hashish, the resin obtained from the cannabis plant (Moreau 1845), a large body of longitudinal studies have accumulated particularly over the last couple of decades investigating the association between cannabis use and the onset of psychotic disorders such as schizophrenia (Andréasson et al. 1987; Arseneault et al. 2002; Bechtold et al. 2016; Ferdinand et al. 2005; Fergusson et al. 2003; Gage et al. 2014; Henquet et al. 2005; Manrique-Garcia et al. 2012; Rognli et al. 2015; Rössler et al. 2012; Van Os et al. 2002; Weiser et al. 2002; Wiles et al. 2006; Zammit et al. 2002). There is growing evidence from studies investigating the neurobiology of psychotic disorders such as schizophrenia as well as those investigating the effects of cannabinoids on brain function that there is substantial overlap in terms of their effects on key neurocognitive substrates involved in the processing of rewarding stimuli and on dopamine, one of the main neurotransmitters involved in signalling reward in the brain (Baik 2013). This is of particular interest as one of the prevailing theories suggests that psychotic symptoms arise in the context of subcortical dopamine dysfunction leading to alteration in the processing of rewarding stimuli resulting in the inappropriate assignment of motivational salience to contextually irrelevant stimuli (Howes and Nour 2016; Kapur 2003).

This narrative review seeks to answer whether the psychotomimetic effects of delta-9-tetrahydrocannabinol (THC) and the antipsychotic potential of cannabidiol (CBD) may be underpinned by the effects on the reward system and dopamine. We synthesise relevant evidence focusing on similarities in altered dopamine signalling and reward processing in people with psychosis and in healthy people who were administered cannabinoids under experimental conditions. Towards this objective, this review will first summarise independent strands of evidence showing an association between psychosis, cannabis use, and dysfunction of the endocannabinoid system. Next, we will summarise key pieces of evidence regarding abnormalities in dopamine signalling and reward processing in psychosis followed by a more detailed focus on the effects of cannabinoid modulation on dopamine signalling and reward processing.

## Methods

In accordance with our objectives, here we identified human studies that examined the neural correlates of the effects of THC and CBD on brain function using acute THC or CBD administration in conjunction with neuroimaging.

### Search strategy

For the purpose of this review, we synthesised human neuroimaging evidence, investigating the acute effects of THC and CBD on brain function with an aim to identify the key brain substrates where THC and CBD have effects.

A systematic search of published human literature was conducted within Ovid MEDLINE, Embase, Global Health, and PsychINFO databases in accordance with the Cochrane Handbook (Chandler et al. 2017) and PRISMA guidelines (Stroup et al. 2000). Two categories of search terms were employed: (1) cannabis, marijuana, marihuana, THC, tetrahydrocannabinol, CBD, cannabidiol and (2) fMRI, functional magnetic resonance, imaging, magnetic resonance, MRI, single -photon emission tomography, SPECT, positron emission tomography, PET, spectroscopy, MRS, perfusion, blood flow, ASL. Categories were combined utilising Boolean Operator commands. Search of key terms was restricted to titles and abstracts within human studies. Two researchers independently preformed the data search and extraction, with the final literature search being conducted on 29/07/19.

### Eligibility Criteria

Studies were included if they (i) assessed the effect of THC or CBD on brain function using an acute drug challenge paradigm in humans, (ii) used fMRI, positron emission tomography (PET), single-photon emission computed tomography (SPECT) or arterial spin labelling (ASL) to measure brain function, (iii) available in English, and (iv) published in a peer-reviewed journal. Articles that did not assess the effects of THC-rich cannabis extract, THC or CBD on brain function, but primarily assessed the psychological effects, were excluded.

## Cannabis use, endocannabinoid dysfunction, and psychosis

The association between cannabis use and the onset of psychosis has been extensively investigated primarily through longitudinal studies of cannabis users within the general population (Andréasson et al. 1987; Arseneault et al. 2002; Bechtold et al. 2016; Ferdinand et al. 2005; Fergusson et al. 2003; Gage et al. 2014; Henquet et al. 2005; Manrique-Garcia et al. 2012; Rognli et al. 2015; Rössler et al. 2012; Van Os et al. 2002; Wiles et al. 2006; Zammit et al. 2002). Out of a total of 13 studies conducted within this context, 10 reported (Andréasson et al. 1987; Arseneault et al. 2002; Bechtold et al. 2016; Ferdinand et al. 2005; Fergusson et al. 2003; Henquet et al. 2005; Manrique-Garcia et al. 2012; Rognli et al. 2015; Van Os et al. 2002; Zammit et al. 2002) an association between cannabis use and significantly increased risk of developing psychotic symptoms or a schizophrenia-like psychotic illness. The remaining three studies showed a trend in the same direction but did not reach statistical significance (Gage et al. 2014; Rössler et al. 2012; Wiles et al. 2006). The relationship between cannabis use and an increased risk of psychosis was weaker after subsequently controlling for confounding factors including age, ethnicity, pre-existing psychosis, socioeconomic status, urbanicity, and use of other substances. In some studies, this relationship was attenuated. This is not unexpected as cannabis use is one risk factor, amongst many, that are likely to be associated with an increased risk for psychosis. A recent meta-analysis of pooled data from 10 studies reported on 66,816 study participants examined the magnitude of the association between cannabis use and psychotic outcomes (Marconi et al. 2016). It was reported that relative to non-users, cannabis users (with history of any use) had nearly two times the odds of developing psychotic symptoms or a psychotic disorder, with the risk increasing to nearly fourfold in the heaviest users (Marconi et al. 2016).

A number of studies have also investigated the association between cannabis use and transition to psychosis in individuals at clinical high-risk of developing psychosis (CHR) (Auther et al. 2012; Auther et al. 2015; Corcoran et al. 2008; McHugh et al. 2017; Valmaggia et al. 2014). Whereas some observed a higher risk of transition to psychosis in CHR patients who used cannabis (Auther et al. 2015; McHugh et al. 2017; Valmaggia et al. 2014), other did not find this (Auther et al. 2012; Corcoran et al. 2008). Importantly, a recent meta-analysis found that the pooled relative risk of developing psychosis in CHR individuals following cannabis use was not statistically significant (Farris et al. 2019). Further to the evidence of the association between cannabis use and onset of psychotic illness, a different meta-analysis that pooled data from over 16,500 patients with psychosis identified associations between continued cannabis use and an increased risk of psychotic relapse, hospitalisation, and longer inpatient admission (Schoeler et al. 2016a). Further evidence from first-episode psychosis cohorts indicated that the association between cannabis use and relapse of psychosis persisted even after controlling for socio-demographic and clinical confounders, such as adherence to medication treatment as well as other illicit drug use (Colizzi et al. 2018), with a dose-dependent relationship between use and psychotic relapse (Schoeler et al. 2016b) that was unlikely to be explained by a common genetic factor predisposing to both cannabis use and psychosis (Schoeler et al. 2016b).

Along with the evidence linking cannabis use and relapse of psychosis, work pioneered by Leweke et al. (1999a) first identified alterations in components of the endocannabinoid system, a lipid signalling system involved in the regulation of a number of physiological and homeostatic processes, in people with established psychotic disorders. A body of evidence has accumulated supporting these early findings (De Marchi et al. 2003; Giuffrida et al. 2004; Leweke et al. 2007; Reuter et al. 2017) which have also been identified in patients at clinical-high-risk (Koethe et al. 2009), independent of cannabis use. The most researched endocannabinoids, anandamide and 2-arachidonoylglycerol (2AG), are endogenous ligands for cannabinoid receptors (CB1 and CB2) (Zou and Kumar 2018). In patients with schizophrenia, there have been reports of increased levels of anandamide, relative to healthy controls (De Marchi et al. 2003; Koethe et al. 2019; Reuter et al. 2017). One study further reported a significant decrease of anandamide in patients who entered clinical remission (De Marchi et al. 2003).

Endocannabinoid system alterations have also been identified in cerebrospinal fluid (CSF) (Giuffrida et al. 2004). Relative to healthy controls, elevated CSF anandamide has been reported in the CSF of people with early psychosis, with increased anandamide levels being associated with a delayed transition to psychosis in those in the prodromal phase, indicating a protective role of anandamide in psychosis (Koethe et al. 2009).

Early evidence from positron emission tomography (PET) studies has highlighted increased CB1 availability in patients with schizophrenia relative to healthy controls (Ceccarini et al. 2013; Wong et al. 2010). These findings are in contrast to more recent reports of reduced CB1 availability in patients with established psychosis relative to healthy controls (Borgan et al. 2019; Ranganathan et al. 2016). The results reported by Ceccarini et al. (2013) may be associated with the absence of using arterial blood sampling, which has been reported as necessary to fully quantify the distribution volume of CB1 receptors (Tonietto et al. 2019). Furthermore, the study of Wong et al. (2010) reported higher CB1 levels in the pons; however, this result did not survive correction for multiple comparisons.

Post-mortem evidence is also inconsistent with findings of higher CB1 receptor binding in subjects with schizophrenia relative to healthy controls (Dalton et al. 2011; Dean et al. 2001; Jenko et al. 2012; Newell et al. 2006; Volk et al. 2014; Zavitsanou et al. 2004) and lower or unchanged CB1 receptor mRNA and protein immunoreactivity levels (Eggan et al. 2008; Eggan et al. 2010; Koethe et al. 2007; Urigüen et al. 2009) localised to the frontal cortex. Several factors may have led to the disparate results obtained via the different methods, including condition of the protein, location of the receptor, or specificity of the antibody or radioligand for the receptor (Jenko et al. 2012). Nevertheless, existing evidence generally tends to suggest that endocannabinoid dysfunction may be linked to the pathophysiology of psychotic disorders such as schizophrenia (Leweke et al. 1999a; Leweke et al. 2016; Ranganathan et al. 2016).

While the independent strands of evidence summarised above indicate that altered functioning of the endocannabinoid system, either endogenous or as a result of exposure to recreational cannabis is associated with psychosis, a further line of evidence supporting such a link comes from evidence that cannabinoids like CBD, that target different components of the endocannabinoid system (Zou and Kumar 2018), including as a negative allosteric modulator of CB1 receptors (Laprairie et al. 2015), may have a role in treating psychosis. To date, three randomised clinical trials have been conducted that investigated psychopathological outcomes in patients with an established psychotic disorder following sustained CBD treatment (Boggs et al. 2018; Leweke et al. 2012; McGuire et al. 2018).

The first landmark clinical trial employed a double-blind, randomised, head-to-head comparison between CBD (600 mg) and amisulpride, a potent antipsychotic (Leweke et al. 2012). Both treatment groups reported a significant clinical improvement, but CBD displayed a markedly superior side-effect profile. Moreover, CBD treatment was associated with significantly higher serum anandamide levels, which was significantly associated with clinical improvement. Of note, the CBD dose was reduced from 800 to 600 mg per day as some patients reported unwanted side effects after week 2. Following this study, a secondary exploratory double-blind parallel-group trial was conducted that examined the efficacy of CBD as an antipsychotic in patients with sub-acute schizophrenia randomised in a 1:1 ratio to receive CBD (1000 mg/day; *N* = 43) or placebo (*N* = 45) alongside their existing antipsychotic medication. After 6 weeks of treatment, compared with the placebo group, the CBD group had a small, although statistically significant improvement in the Positive and Negative Syndrome Scale positive scores (1.5 points) (McGuire et al. 2018). The final clinical trial conducted used a 6-week, randomised, placebo-controlled, parallel group, design with 600 mg CBD or placebo in patients diagnosed with chronic schizophrenia (although patients continued taking any existing psychopharmacological treatment) (Boggs et al. 2018). This study reported no significant effect of CBD on patient symptoms, relative to placebo; although 600 mg has been shown to attenuate psychosis-like effects in acute laboratory studies (Bhattacharyya et al. 2010), a higher dose may be required to produce beneficial effects on psychotic symptoms in chronic schizophrenia. The stage of the illness should also be considered, as it may be possible that CBD is more effective during early psychosis where patients have also demonstrated alterations in endocannabinoid levels (Koethe et al. 2009; Leweke et al. 2012).

## Dopamine, reward processing, and psychosis

Although the association between dopamine dysfunction and psychosis is well-known, it was only the fortuitous discovery of early antipsychotic agents in the 1950s that led to a focus on alterations in dopamine neurotransmission as an underlying abnormality in psychosis (Tost et al. 2010). Radio-ligand-based neuroimaging techniques such as positron emission tomography (PET) and single-photon emission computed tomography (SPECT) have since provided support that increased striatal presynaptic dopamine synthesis and release may be involved in the pathology of psychosis (Fusar-Poli and Meyer-Lindenberg 2013; Ginovart et al. 2004; Howes et al. 2012).

It has been observed repeatedly that patients with established psychosis assign greater attention and significance to irrelevant or neutral stimuli (Chapman 1966; McGhie and Chapman 1961). This has been hypothesised to arise from increases in spontaneous dopaminergic firing in mesolimbic reward pathways leading to abnormal stimulus-reinforcement generating the onset of psychotic symptoms (Miller 1976). Later termed, ‘aberrant salience’, this theory postulates that psychosis may arise from the inappropriate assignment of salience to contextually irrelevant external cues (Kapur 2003). In this theory, salience refers to the motivational component of a stimulus which captures attention mediated by ventral striatal dopaminergic release (Berridge and Robinson 1998).

In this review we discuss salience (and prediction errors) within the context of reward. There is general consensus that the processing of rewards involves functions including the signalling of mismatches between received and predicted rewards (termed reward prediction error), which facilitates learning, and the attribution of salience to stimuli and outcomes that are particularly noticeable. We are particularly interested in these functions as they have been proposed to underpin the symptoms of psychosis.

Predicting which actions are associated with the highest reward (i.e. best outcome) is crucial for effective decision making and adaptive behaviour (Diederen and Fletcher 2020). An important signal for learning to predict future outcomes is the prediction error (PE) which is the expected outcome vs the outcome received (Schultz 2016b). Whereas, PEs occur across sensorimotor and value domains (Den Ouden et al. 2012), reward PEs (RPEs), do not only signal the extent of the mismatch between outcomes and predictions, but also indicate whether outcomes were better or worse than expected, resulting in positively and negatively signed PEs (Den Ouden et al. 2012).

The mesolimbic pathway facilitates RPE signalling and relays dopamine from the ventral tegmental area (VTA) to the nucleus accumbens (NA) in the ventral striatum (García-García et al. 2017). In addition, the nigrostriatal pathway connects the pars compacta to the dorsolateral striatum, and the premotor/motor cortex, which has been hypothesised to be involved in action selection, facilitating the acquisition of maximal reward (García-García et al. 2017). Further to its function in RPE coding, there is some evidence that suggests that dopamine codes salience, that is, the extent that a stimulus, outcome or action, is particularly noticeable (Schultz 2016a). It is important to note that RPE coding and salience are not mutually exclusive as the experience of any type of PE, including RPEs, is salient (Diederen and Fletcher 2020). However, some work has suggested that dopaminergic responses to salience and RPEs occur at different timescales (Schultz 2016a).

Different types of salience have been defined, including motivational salience. Motivational salience refers to approach-guided behaviour for rewarding outcomes (also referred to as incentive salience), and the avoidance of aversive outcomes once a stimulus has been processed (Robinson and Berridge 2008). The attribution of motivational salience is thought to occur in the time between the reward has been identified, and the action to pursue it (McClure et al. 2003). It has been reported that four types of value-sensitive neurons are present which correspond to reward-ON, reward-OFF, aversive-ON, and aversive-OFF, and only reward-ON may be dopamine-mediated (Fiorillo 2013).

In line with this observation, it has long been hypothesised that dopamine responds selectively to positively balanced (i.e. rewarding) outcomes (Robinson and Berridge 2008). Robinson and Berridge (2008) further suggest that mesolimbic dopamine is selectively involved in attributing salience to guide approach behaviour, and that it has no role in RPE coding. Specifically, the authors propose that inhibiting dopamine selectively prevents reward-seeking behaviours, without altering valuation and the associated RPE of an outcome. This is in strong contrast to alternative bodies of evidence for dopamine in RPE coding, and it has been proposed that dopamine in fact holds a dual role, involved in learning from RPEs and ongoing approach behaviour (McClure et al. 2003; Schultz 2016a).

Aberrant salience attribution has been associated with abnormal prediction error processing, formulating the association between reinforcement learning abnormalities and psychotic symptoms (Diederen and Fletcher 2020; Fletcher and Frith 2009; Heinz and Schlagenhauf 2010). It has been hypothesised that the number of dopaminergic neurons involved in relaying prediction error signals may be upregulated in schizophrenia, contributing to abnormal salience processing (Lodge and Grace 2011). There are however, other explanations of how altered prediction error signalling might result in psychotic symptoms (Maia and Frank 2017; Valton et al. 2017).

Neuroimaging studies have provided evidence of impaired reward processing and abnormal engagement of the striatum in established psychotic disorders. A meta-analysis of functional magnetic resonance imaging (fMRI) studies using reward-based paradigms such as the monetary incentive delay and reward prediction error learning tasks has reported reduced bilateral ventral striatal activation during the anticipation of reward in psychosis, suggesting altered processing of salient reward-predicting stimuli (Radua et al. 2015). These findings are in line with the original aberrant salience theory suggesting that healthy people are commonly associated with dopamine release prior to receiving hedonic outcomes, seen in the anticipatory phase (Kapur 2003). Interestingly, functional connectivity analysis has revealed a putative salience network (Seeley et al. 2007) involved in the choice of directing attention to stimuli from a continuous stream of internally and externally generated inputs to the anterior cingulate and anterior insula (Uddin 2015), regions found to be implicated in the neuropathology of psychosis.

Preliminary studies examining neural activation and reward processing in patients at different stages of psychosis have reported intriguing results. First episode psychosis patients have been shown to have abnormal meso-cortical signalling of reward-prediction errors (Ermakova et al. 2018). Patients who are clinical at high risk (CHR) of developing psychosis have a more nuanced pattern of activation with a degree of midbrain impairment but preserved cortical function (Ermakova et al. 2018; Haarsma et al. 2020). Medicated patients with established psychosis present with blunted neural responses for positive reward prediction errors in the striatum, midbrain, and other limbic regions, which have correlated with negative symptoms (Maia and Frank 2017). Furthermore, unmedicated patients present with blunted ventral striatal and midbrain activity for prediction errors (Maia and Frank 2017). While further research is required to provide a better understanding of the nature of alterations and how they may be related to the psychopathology of psychosis, robust evidence exists in terms of altered reward processing and dysfunction of neural substrates with rich dopaminergic inputs in psychosis (Radua et al. 2015).

Therefore, evidence suggests that RPE and salience signalling are associated with dopaminergic firing within the reward-based circuitry of the brain, which have been specifically observed as abnormal in patients with psychosis, giving rise to the aberrant salience hypothesis. Thus, the underlying psychotomimetic and putative antipsychotic effects of THC and CBD respectively may also be related to RPE and salience processing. Current evidence on the effects of cannabinoids on specific areas of reward are limited; thus, future investigation is required using comprehensive reward-based models to examine RPE and salience mechanisms in cannabinoid drug challenges.

## Cannabinoids, dopamine, and reward processing

Understanding the effect of cannabis use on alterations in dopamine and reward processing is challenging in the context of observational studies, not just because of potential alternative explanations that may usually confound any observed association between exposure and change in candidate mechanistic substrates in general, but because of heterogeneity in the content of the exposure itself that poses a unique challenge in the case of cannabis. Of foremost importance, recreational cannabis is not one substance; the extract of Cannabis sativa has been reported to contain over 100 different phytocannabinoids, with THC and CBD being the most abundant (Thomas and ElSohly 2016). Given the relatively modest amounts of cannabinoids other than THC and CBD in recreational cannabis, observational studies remain informative about the more general effects of cannabinoids such as THC and CBD. The effects of these different phytocannabinoids may potentially have disparate and often unknown effects on the brain and behaviour; however, further research is required to investigate their effects. Early investigative research identified the psychotomimetic properties of THC in humans (Isbell et al. 1967; Leweke et al. 1999b; Melges 1976). It was later identified in a double-blind placebo-controlled study, administering 2.5 and 5 mg of THC intravenously, that a dose-dependent relationship existed between THC and transient psychotic symptoms induced (D’Souza et al. 2004). It has been suggested that CBD may ameliorate psychotic-like symptoms induced by THC (Morgan and Curran 2008). These findings have been supported by an early study that investigated the presence or absence of CBD and high versus low levels of THC (in human hair) on psychotic like symptoms (Morgan et al. 2012). Two further randomised, placebo controlled clinical trials report that CBD pre-treatment may prevent the acute induction of psychotic symptoms by THC (Bhattacharyya et al. 2010; Englund et al. 2013).

The study of Englund et al., (2013) reported a reduction in positive psychotic symptoms following 1.5 mg intravenous THC, which did not reach significance following pre-treatment of 600 mg oral CBD. However, a significant protective effect of CBD was found when the authors compared the number of people who met the criteria for clinically significant psychosis (an increase from baseline of ≥ 3 points). Contrary to these findings, two separate studies have reported that THC, when administered with CBD, did not protect against the acute psychotic-like effects of cannabis when compared with THC without CBD (Mokrysz et al. 2020; Morgan et al. 2018). These differences in results may be associated with the considerably larger CBD dosage administered by Englund et al. (2013) who used 600 mg relative to Morgan et al. (2018) who used 16 mg, and Mokrysz et al. (2020) who used 10 mg. Considering the different routes of administration, the dosages are not directly comparable; however, Englund et al. (2013) may have achieved a greater yield of CBD absorption, accounting for the difference in results. Finally, it may be possible that the protective effects of CBD are more pronounced in those who are already sensitive to the psychotomimetic effects of THC.

Of note, one study has reported subjective intoxication following 400 mg vaporised CBD, relative to placebo (Solowij et al. 2019). Solowij and colleagues reported that subjective intoxication under the influence of CBD manifested as a dissociated state, which correlated with the depersonalisation and derealisation scores on the Clinician Administered Dissociative States Scale (CADSS), in addition to the CADSS total score, but not the amnesia subscale. Further correlations were observed with the Visual Analogue internal and external perception scales, but not with drowsiness. The sedating effects of CBD is consistent with the findings of other studies (Russo and Guy 2006; Zuardi et al. 2012). Moreover, Solowij et al. (2019) reported a correlation between independent observer ratings of intoxication with participant ratings of drowsiness following drug administration, participant ratings of changes in external perception, and at trend level internal perception and CADSS total score. These findings indicate that observer ratings of intoxication may have been predicated on the perception of the participants’ drowsiness and behaviours, suggesting that the participants were responding differently to their external environment and dissociating. Solowij’s study suggests that CBD may have intoxicating effects, which include feelings of depersonalisation, derealisation, and altered internal and external perceptions. However, it was reported that the CBD condition induced significant coughing. Together with the relative ease of inhaling ethanol-flavoured air in the placebo condition compared to the CBD condition, this may have inadvertently caused unblinding of subjects. The changes in intoxication reported under CBD condition may therefore, have been related to expectation bias (Solowij et al. 2019). Hence, any conclusion about subjective effects of CBD on the basis of one study is premature. Nevertheless, we cannot rule out the possibility that CBD may have some intoxicating properties.

## Pre-clinical evidence of cannabinoid modulation of dopamine function

In order to investigate the effects of cannabinoids on dopamine, early studies were conducted in animals. The landmark paper by Giuffrida et al. (1999) first suggested that a functional interaction may exist between endocannabinoids and dopaminergic systems, in contributing to striatal signalling. The study measured anandamide release in the dorsal striatum of freely moving rats using microdialysis and gas chromatography/mass spectrometry. Anandamide release was eight times higher than baseline following local administration of the D2-like dopamine receptor agonist quinpirole, a response that was prevented by the D2-like receptor antagonist raclopride. Administration of the D1-like receptor agonist SKF38393 had no such effect (Giuffrida et al. 1999).

Pre-clinical studies have since suggested that there is involvement of the endocannabinoid system during reward processing specifically (Solinas et al. 2007). THC has been associated with increases in dopamine neurotransmission in the mesolimbic dopamine system (Kolb et al. 2006; Oleson and Cheer 2012), specifically the nucleus accumbens shell (Tanda et al. 1997). Studies using microdialysis have identified that cannabinoid CB1 agonists (such as THC, a partial agonist (Pertwee 2008)) increases dopamine levels in the nucleus accumbens (Braida et al. 2001; Chen et al. 1990; Chen et al. 1991; Chen et al. 1993), specifically its shell sub-region (Tanda et al. 1997). While dopamine elevations within the nucleus accumbens are not a direct measure of reward, they are an important neurochemical correlate of reward and a potential mechanism for the effects seen by cannabinoids (Solinas et al. 2007b). Dopaminergic firing within the ventral tegmental area has also been increased by CB1 agonists in rodents (French et al. 1997; French 1997; Gessa et al. 1998; Melis et al. 2000). Furthermore, using the dopamine transporter (DAT) knockout model of schizophrenia in mice (Giros et al. 1996; Hill and Tasker 2012) characterised by hyperdopaminergia primarily within the striatum and nucleus accumbens (Kasahara et al. 2013), it has been shown that DAT knockout mice present with reduced levels of the endocannabinoid, anandamide within the striatum (Tzavara et al. 2006). In contrast, repeated THC administration, a known risk factor for psychosis, has been shown to downregulate anandamide in the striatum (Di Marzo et al. 2000).

**Fig. 1:**
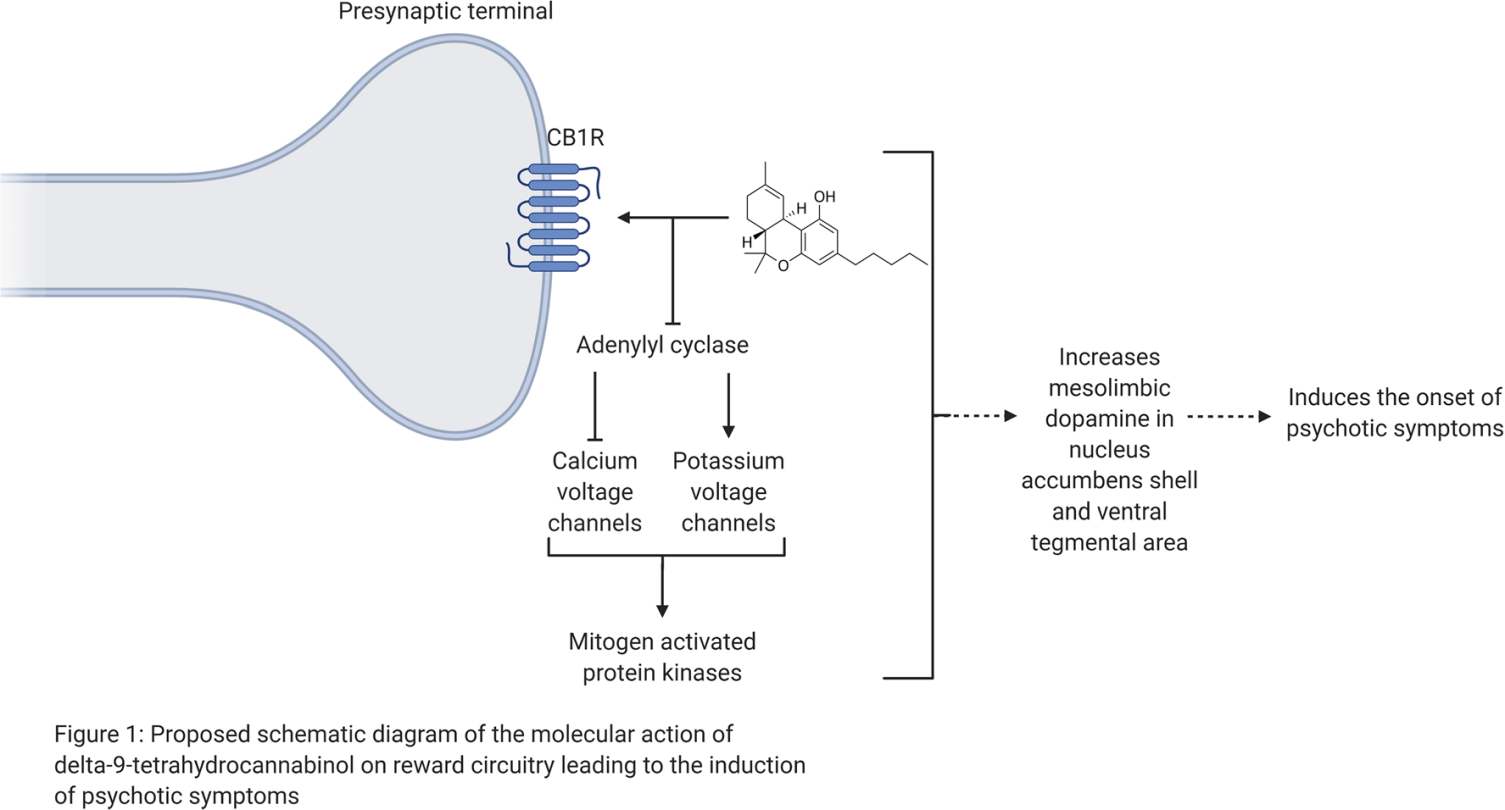
Proposed schematic diagram of the molecular action of delta-9-tetrahydrocannabinol on reward circuitry leading to the induction of psychotic symptoms

Other reviews have focused on the molecular action of THC on the reward circuitry of the brain (Scherma et al. 2019), which are not the focus here. Nevertheless, we detail relevant evidence on the molecular action of THC and CBD. Both CB1R and CB2R are G protein-coupled receptors (GPCRs). Anandamide, 2-AG, and THC have a high affinity in binding to CB1 receptors. CB1 receptors are the most abundant GPCRs in the brain, rich in areas including the striatum, parahippocampus, anterior cingulate/medial prefrontal cortex, and amygdala (Mackie 2005). CB1 receptors are primarily expressed on axons and axon terminals of neurons, but also on interneurons and astrocytes (Breivogel et al. 1998; Breivogel and Childers 1998). Following CB1 receptor activation, its mechanism involves a signalling cascade that leads to the inhibition of adenylyl cyclase (Howlett 1985), inhibition of the opening of voltage-gated calcium channels (Mackie and Hille 1992), an increase in potassium channel conductance (Deadwyler et al. 1995; Mackie and Hille 1992), and activation of the mitogen-activated protein kinases (MAPKs) (Bouaboula et al. 1995) (Fig. 1). In contrast to the traditional view that CB2 receptors are limited in expression of the immune system (Munro et al. 1993), recent reports have identified CB2 receptor expression in midbrain DA neurons (Liu et al. 2017; Zhang et al. 2017). Here, CB2 receptors modulate alcohol preference and the reinforcing and neurochemical effects of cocaine (Ishiguro et al. 2007; Xi et al. 2011; Zhang et al. 2014). However, species differences related to CB2R genes splicing and their functional expression may lead to different behavioural outcomes related to cocaine self-administration (Scherma et al. 2019). In addition, CB1 and CB2 receptors differ in their signalling; CB2 receptors poorly modulate calcium channels and inwardly rectifying potassium channels (Felder et al. 1996). Moreover, CB2 receptors from different species report varying pharmacological results in response to activation by identical drugs (Bingham et al. 2007; Yao et al. 2006).

The mesocorticolimbic system is composed of subpopulations of dopaminergic neurons that originate from the ventral tegmental area (VTA) and pars compacta of the substantia nigra. These neurons project to the nucleus accumbens and other limbic structures. The mesocorticolimbic system is a critical component of reward processing and motivated behaviour (Fields 2007; Wise 2009; Wise and Rompre 1989). Mesocorticolimbic dopaminergic neurons are mediated by excitatory (primarily glutamatergic) and inhibitory (primarily GABAergic) inputs that regulate their neuronal activity (Kalivas 1993; Korotkova et al. 2004; Morales and Root 2014; Wise and Morales 2010). Further to these reports, there is evidence that the endocannabinoid system is involved in the neurocognitive process of reward in modulating dopaminergic neurons (Bloomfield et al. 2016; Solinas et al. 2006; Solinas et al. 2007a).

CB1 receptors are abundant in the VTA, nucleus accumbens, prefrontal cortex, central amygdala, and hippocampus where they are primarily located at the presynaptic terminal (Herkenham et al. 1991). Once activated, CB1 receptors function as retrograde messengers which inhibit neurotransmitter release (Schlicker and Kathmann 2001; Wilson and Nicoll 2001). Therefore, endocannabinoids are released in both the nucleus accumbens and the VTA following depolarisation (Melis et al. 2004; Robbe et al. 2002). CB1 receptors on the axon terminals of GABAergic neurons in the VTA, and glutamatergic neurons in both the VTA and nucleus accumbens have been shown to inhibit neurotransmission once activated (Lupica and Riegel 2005; Melis et al. 2004). In addition, the final effect on VTA dopaminergic activity is dependent on the level of relative input activation influenced by varying behavioural circumstances (Lupica and Riegel 2005).

Rewarding stimuli prompt dopamine release in the nucleus accumbens shell (Bassareo and Di Chiara 1997; Martel and Fantino 1996; Tanda and Di Chiara 1998). AEA, 2-AG, and THC also increase extracellular dopamine levels in the nucleus accumbens shell, indicating that they may have a role in reward, or reward reinforcement of (De Luca et al. 2014; Solinas et al. 2006; Tanda et al. 1997). Significantly, this observation is prevented following administration of rimonabant, a CB1 antagonist. These findings suggest that the dopaminergic effects of endocannabinoids involve CB1 receptors (Solinas et al. 2006; Tanda et al. 1997). Further evidence stems from the reduction, or inhibition, of transient dopamine increases following the administration of rewarding stimuli (such as drugs, cocaine, ethanol, and nicotine, or palatable food) in the nucleus accumbens shell when CB1 receptors are blocked using a pharmacological intervention in rats (Cheer et al. 2007; Melis et al. 2007). In concurrence with these findings, other studies have reported altered levels of AEA and 2-AG in the presence of rewarding stimuli following drug administration in regions including the limbic forebrain, striatum, and hippocampus, albeit in varying directions (Caillé et al. 2007; Centonze et al. 2004; González et al. 2002; Thiemann et al. 2008; Viganò et al. 2004).

**Fig. 2:**
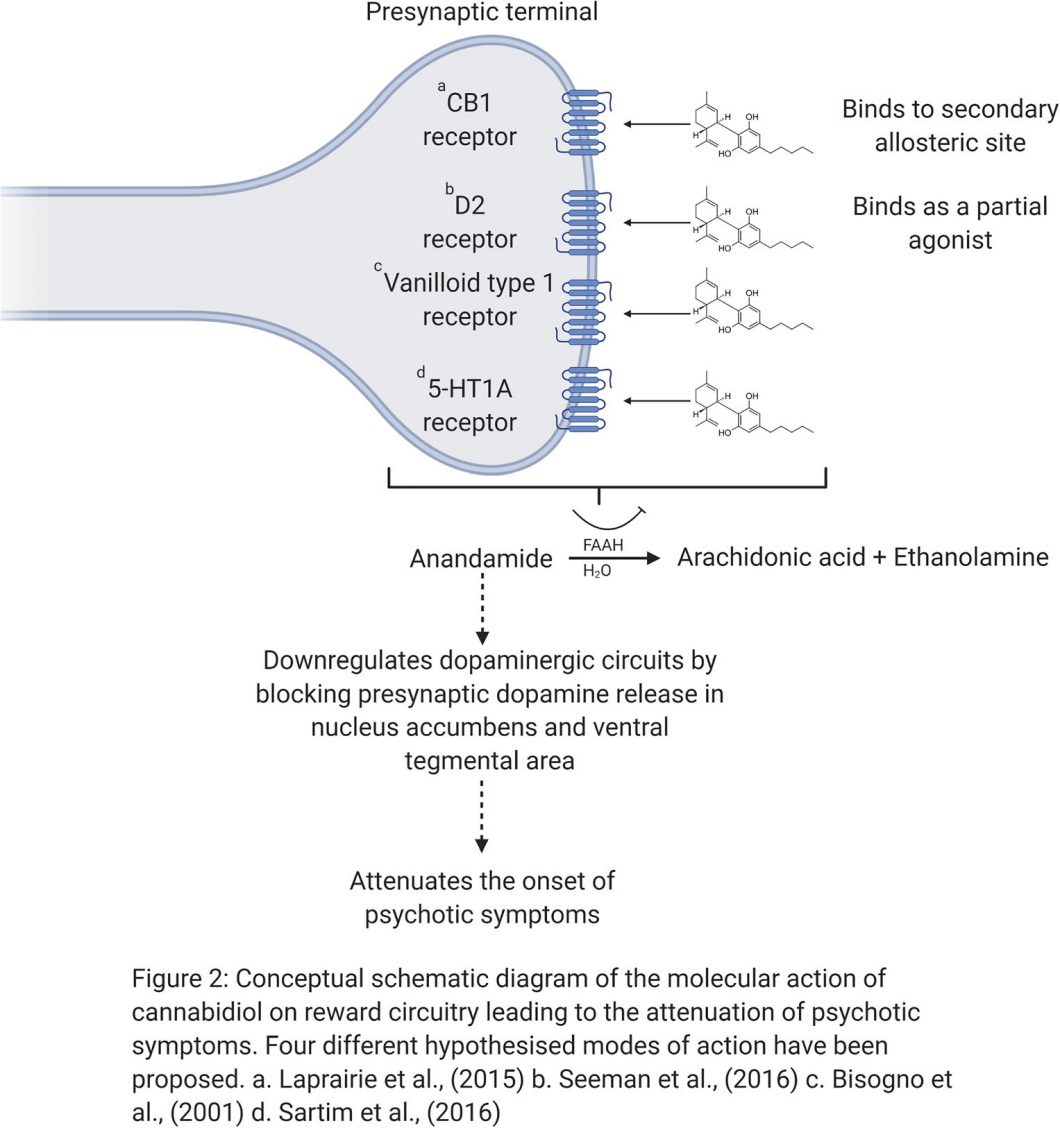
Conceptual schematic diagram of the molecular action of cannabidiol on reward circuitry leading to the attenuation of psychotic symptoms. Four different hypothesised modes of action have been proposed. (a) Laprairie et al. 2015. (b) Seeman (2016). (c) Bisogno et al. (2001). (d) Sartim et al. (2016)

CBD, on the other hand, possesses a more diverse pharmacological profile. Following CBD administration, there have been reports of negative allosteric modulation of CB1 receptors with weak antagonism of CB2 receptors, partial agonism of D2 receptors, inhibition of anandamide hydrolysis, and stimulate vanilloid receptor type 1 and 5-HT1A receptors (5-HT1AR) (Bisogno et al. 2001; Laprairie et al. 2015; Sartim et al. 2016). Little is known about the effects of CBD on the mesolimbic system, particularly on dopaminergic function. Biochemical studies suggest that CBD may enhance endogenous anandamide signalling indirectly, by inhibiting the intracellular degradation of anandamide catalysed by the enzyme fatty acid amide hydrolase (FAAH) in rats (Bisogno et al. 2001) or fatty acid-binding proteins (FABP) in humans (Elmes et al. 2015). This may in turn downregulate dopaminergic circuits by blocking presynaptic dopamine release (Giuffrida et al. 1999), an effect very much in line with respective clinical observations (Leweke et al. 2012) (Fig. 2). Amphetamine dopaminergic sensitisation has been employed to model the development of aberrant mesolimbic dopamine signalling and the subsequent dysregulation of incentive motivational processes (O’Daly et al. 2014). Using this model, CBD administration has been shown to target the nucleus accumbens shell and attenuate amphetamine-induced sensitisation, in terms of psychotomimetic behaviours (hyperlocomotion and sensoriomotor-gating deficits) and dopaminergic neuronal activity within the ventral tegmental area (Renard et al. 2016). This model highlights promise as to the potential mode of CBD action as, the nucleus accumbens shell is also the target of most effective antipsychotics (Ananth et al. 2001). Importantly, all current antipsychotics act by interfering with the action of dopamine at dopamine D2 receptors (Li et al. 2016).

A separate study utilised tritiated domperidone to label rat brain striatal D2 receptors (Seeman 2016). It was reported that CBD inhibited the binding of radio-domperidone, with dissociation constants of 11 nM at dopamine D2High receptors and 2800 nM at dopamine D2Low receptors. This biphasic mechanism has also been observed in aripiprazole, a dopamine partial agonist antipsychotic drug. The clinical doses of CBD were sufficient to occupy the functional D2High sites; thus, it was concluded that the dopamine partial agonist action of CBD may account for its clinical antipsychotic effects (Seeman 2016). Other evidence suggests that CB1 antagonists (such as CBD (Pertwee 2008)) may function by directly or indirectly blocking the effects of THC or other psychoactive drugs on dopaminergic function or signalling downstream of dopamine receptors (Galaj et al. 2020; Hudson et al. 2019; Renard et al. 2016).

## Human evidence of acute cannabinoid modulation of dopamine function and reward processing

Although the role of THC in increasing synaptic dopamine levels in the striatum and its rewarding effects have been observed pre-clinically, effects in humans have been less clear. Experimental studies have demonstrated that in healthy individuals, transient psychotic symptoms may be induced following THC administration (D’Souza et al. 2004; Isbell et al. 1967; Leweke et al. 1999b; Melges 1976). The psychotomimetic effects of THC have been correlated with functional activation of brain regions commonly implicated in psychosis, particularly striatal regions (Bhattacharyya et al. 2009; Bhattacharyya et al. 2012b; Bhattacharyya et al. 2012a). Pooled results (Bossong et al. 2015) from two separate PET studies (Bossong et al. 2009; Stokes et al. 2009) using the radiotracer [^11^C]raclopride identified significant reduction of radiotracer binding in the limbic striatum, consistent with increased dopamine levels in this region following THC administration relative to placebo, suggesting that increased striatal dopamine levels may be a mechanism underlying psychotic symptoms induced by THC (Bossong et al. 2015). However, the initial PET studies (Bossong et al. 2009; Stokes et al. 2009) had contradictory findings, and the effect of THC on striatal dopamine levels have not been replicated in another study (Barkus et al. 2011). In light of the absence of replication, it is important to consider the results reported by Bossong et al. (2015) in the context of the size of the effect of THC relative to other recreational drugs in inducing striatal dopamine release. Amphetamine and alcohol have been observed to reduce limbic striatal dopamine D2/D3 receptor availability in the range of 10 to 15% (Boileau et al. 2003; Martinez et al. 2003; Martinez et al. 2007; Oswald et al. 2005; Urban et al. 2010), while nicotine has also been reported to reduce limbic striatal availability by approximately 10% (Brody et al. 2009; Takahashi et al. 2008). Therefore, the modest effect of THC in modulating striatal dopamine release (as indexed by dopamine D2/D3 receptor availability) despite being associated with robust behavioural effects may suggest that cannabinoid modulation of dopamine signalling may not fully explain the acute psychotomimetic effects of THC, especially in light of early evidence of alternative mechanisms such as effects on the glutamatergic system (Colizzi et al. 2019; Mason et al. 2019).

Building upon the results from PET studies, one study has also investigated the effect of acute THC challenge compared to placebo on reward processing in healthy participants using the monetary incentive delay task (MIDT) (Van Hell et al. 2012) in conjunction with fMRI. Reaction time was slower in responding to stimuli under THC compared to the placebo condition, suggesting the presence of a motor or attentional effect. Using a region of interest analysis approach focusing on the task network engaged under the two drug conditions, this study showed that THC administration attenuated brain activity during reward feedback, largely seen in the inferior parietal and temporal cortices (Van Hell et al. 2012), but did not have any significant effect on activation during the anticipation phase. However, the study was underpowered with only 11 subjects in the final analysis with a relatively low dose of THC (6 mg) administered via inhalation route that resulted in peak blood levels of THC that were much lower than that attained in other studies that have shown either an effect of THC on dopamine (Bossong et al. 2009) or glutamate levels (Colizzi et al. 2019). Furthermore, this study did not specifically measure the acute psychotomimetic effects of THC limiting their ability to examine the association of such effects with the effects of THC on the brain. Although, reaction time was slower in responding to all stimuli under THC compared to the placebo condition, with a more pronounced effect during rewarding stimuli, the relationship between neural and behavioural effects was not examined, a behavioural correlate of particular interest in this context in light of evidence of direct correlation between motivational salience of stimuli and reaction time (Mir et al. 2011), as well as independent evidence of effect of THC and cannabis on salience attribution (Wijayendran et al. 2018). Of note, a secondary report from the same cohort of subjects was conducted of those with nicotine addiction. In using a region of interest analysis approach that focused on nucleus accumbens, caudate, and putamen, attenuation of activation was observed in the nucleus accumbens following THC relative to placebo during the anticipatory phase of the MIDT (Jansma et al. 2013).

To investigate the interactive effects of cannabinoids on reward, a randomised, crossover, placebo-controlled, double blind investigation was conducted in healthy volunteers (Freeman et al. 2018; Lawn et al. 2016). Vaporised cannabis with CBD (Cann+CBD) and without CBD (Cann−CBD) were investigated in the context of effort-related decision-making (Lawn et al. 2016). Cann−CBD led to a lower likelihood of making a high-effort choice (i.e. motivation) for monetary reward than placebo, but there was no difference between Cann−CBD and Cann+CBD. Moreover, Cann−CBD increased sensitivity to expected value of the monetary outcomes, relative to both placebo and Cann+CBD. Therefore, the results presented here indicate that acute cannabis may induce transient amotivation, and CBD may offset the effects of THC in reducing motivational salience.

A subsequent report from the same study investigated the interactive effects of cannabinoids during a fMRI musical listening paradigm, relative to scrambled sound (Freeman et al. 2018). The effects of Cann+CBD were compared with Cann−CBD. This study is significant as cannabis has been previously reported to dampen response to music in several regions implicated in music-evoked reward and emotion (Koelsch 2014). Across all scans, a positive correlation was reported between response to music in the ventral striatum and the pleasure of listening to the same sound clips, consistent with other studies implicating the ventral striatum in musical pleasure (Blood and Zatorre 2001; Koelsch et al. 2006; Menon and Levitin 2005; Salimpoor et al. 2011; Trost et al. 2012). Following administration of Cann−CBD, relative to placebo, attenuation of regions including the superior temporal gyrus, planum temporale, hippocampus, amygdala, and ventral striatum was observed. When comparing Cann+CBD with placebo, or Cann+CBD with Cann−CBD, no significant differences in activation were reported. The findings reported by Freeman et al. (2018) are in line with a 4-year prospective study that reported an association between increased cannabis and reductions in ventral striatal response to reward anticipation (Martz et al. 2016).

Aside from this, evidence from other studies that have not used a reward processing task specifically in the context of fMRI (Batalla et al. 2014; Bhattacharyya et al. 2012a) also indicate that an acute dosage of THC may modulate key regions involved in reward and salience processing such as the striatum, midbrain, insula, and anterior cingulate cortex. Following THC administration, relative to placebo, studies have reported both increases (Atakan et al. 2013; Bhattacharyya et al. 2014b; Bhattacharyya et al. 2014a; Borgwardt et al. 2008) and attenuation of striatal activation (Bhattacharyya et al. 2009; Bhattacharyya et al. 2012b; Freeman et al. 2018). Furthermore, the effects of THC on the striatum have been correlated with the severity of acute psychotic symptoms induced under its influence (Bhattacharyya et al. 2009; Bhattacharyya et al. 2012b). In the insula, a number of BOLD fMRI studies have reported attenuation of activation under THC compared to placebo (Battistella et al. 2013; Bhattacharyya et al. 2012b; Bhattacharyya et al. 2014b; Bhattacharyya et al. 2014a; Bossong et al. 2012a; Winton-Brown et al. 2011) while another study has reported THC-induced increases in activation (Bhattacharyya et al. 2017). Mixed results have been reported in the anterior cingulate cortex. Decreases (Bhattacharyya et al. 2009; Borgwardt et al. 2008; Rabinak et al. 2012) as well as increases (Battistella et al. 2013) in activation have been observed following THC when compared with placebo. On the other hand, a number of studies that employed a region-of-interest (ROI) analysis approach to investigate the effects of THC on these regions did not find significant activation of the insula (Bossong et al. 2012b; Van Hell et al. 2012; Walter et al. 2017) or of the anterior cingulate (Bossong et al. 2013; Freeman et al. 2018; Hammoud et al. 2019; Van Hell et al. 2012). It is important to note that the study of Van Hell et al. (2012) employed the monetary incentive delay task, a task designed to specifically model reward. The results discussed here may indicate that THC modulates the functioning of the brain regions mentioned above, irrespective of the cognitive task employed. Therefore, the action of THC may be related to its pharmacological effect on CB1 receptors, the main molecular target of THC in the brain, especially when considering the relative density of CB1 receptors in these regions (Herkenham et al. 1991). Given that these regions have a role in reward and salience processing, effect of THC on these neural substrates irrespective of the cognitive task employed may indirectly point towards the potential for THC to modulate reward/salience processing, perhaps through an effect on these regions.

While cannabinoid research has mainly focused on the effects of THC, there is additional evidence regarding the effect of CBD during reward processing, that is especially pertinent given that CBD appears to have an antipsychotic effect (Leweke et al. 2012; McGuire et al. 2018), which contrasts the psychotomimetic effects of THC. To elucidate the neural substrates involved in the antipsychotic effect of CBD, one study investigated the effect of CBD on the brain substrates involved in the context of reward processing. In a CHR population using the MIDT (*n* = 33), this study (Wilson et al. 2019) identified increased activation in the left insula/parietal operculum in CHR patients under placebo condition compared to healthy controls that was correlated with both positive psychotic symptoms. The insular cortex, along with the anterior cingulate cortex is a key component of the ‘salience network’, involved in the processing of motivational salient stimuli (Seeley et al. 2007; Uddin 2015). A single dose of CBD attenuated the increased activation in the left insula/parietal operculum in CHR patients (Wilson et al. 2019), further indicating that cannabinoids such as CBD may also modulate key components of the reward processing network. Although this study has been the only investigation carried out using a reward-based paradigm, other studies employing a non-reward (verbal memory) fMRI paradigm have also shown that a single dose of CBD may modulate the function or connectivity of the striatum and midbrain (Bhattacharyya et al. 2018; O’Neill et al. 2020), key dopaminergic brain regions that are also involved in the processing of motivationally salient stimuli.

Further evidence from studies in healthy volunteers indicate that CBD has opposing effects to that of THC on the function and connectivity of brain regions involved in reward and salience processing such as the striatum (Bhattacharyya et al. 2010; Bhattacharyya et al. 2012b; Bhattacharyya et al. 2015; Winton-Brown et al. 2011). In different parts of the striatum, CBD has consistently enhanced brain activity, while THC has consistently decreased regional activation during the same cognitive paradigms (Bhattacharyya et al. 2010; Bhattacharyya et al. 2012b). Fewer studies have examined the opposing effects of these cannabinoids on the functional integration between different brain regions. One study resting-state study has reported an increase in connectivity from a dorsal striatal seed to the inferior frontal gyrus following CBD, and reduced connectivity following THC, relative to placebo (Bhattacharyya et al. 2015). The same study also reported an increase in connectivity from a hippocampal seed to anterior cingulate cortex following THC relative to placebo, and a reduction in connectivity following CBD compared with placebo (Bhattacharyya et al. 2015).

A consistent opposite pattern of effect in these regions across a range of cognitive paradigms may indicate that these effects are not necessarily a task-specific effect but may reflect a more general pharmacological effect on the blood oxygen level-dependent (BOLD) haemodynamic response signal in these brain regions. Furthermore, the effects of THC on the BOLD signal in the striatum during two different cognitive tasks (memory and attentional salience) inversely correlated with the level of psychotic symptoms induced by it (Bhattacharyya et al. 2010). These findings are consistent with the psychotomimetic effects of THC (Solowij 2018) and the proposed antipsychotic properties of CBD (Batalla et al. 2019).

Piecing these findings together, the above studies suggest that increases in spontaneous dopaminergic firing in mesolimbic reward pathways may underlie the onset of psychotic symptoms (Miller 1976), potentially through altered function in (and connectivity between) the hippocampus, ventral striatum, and the midbrain (Radua et al. 2015; Winton-Brown et al. 2017). These regions have been shown to be robustly modulated by THC across a number of neurocognitive paradigms (Battistella et al. 2013; Bhattacharyya et al. 2009; Bhattacharyya et al. 2012a; Bhattacharyya et al. 2017; Borgwardt et al. 2008; Bossong et al. 2012a; Freeman et al. 2018), with the effects of THC within striatal regions correlated with the severity of acute psychotic symptoms induced under its influence (Bhattacharyya et al. 2009; Bhattacharyya et al. 2012b; Bhattacharyya et al. 2012a). Conversely, in patients with psychosis and those at high-risk, acute CBD has been observed to modulate the activation and connectivity of the medial temporal cortex, midbrain, and striatum, such that functional abnormalities in these regions relative to healthy controls are less under its influence than under placebo (Bhattacharyya et al. 2018; O’Neill et al. 2020). Collectively, opposing effects of THC and CBD in healthy individuals, as summarised here, are consistent with evidence of the role of the identified regions in psychosis, the psychotogenic and potential antipsychotic effects of THC and CBD, respectively, as well as the mechanisms that may underlie the antipsychotic effects of CBD in clinical populations.

## Complementary evidence of dopamine function alteration in cannabis users

Complementary evidence from PET studies provided additional insight into cannabinoid-induced dopaminergic alterations in human cannabis users. Chronic cannabis use has been associated with reduced striatal dopamine synthesis capacity (Bloomfield et al. 2014) that correlated with the extent of cannabis consumption (Bloomfield et al. 2014). Two independent studies have further demonstrated reduced striatal dopamine release within cannabis users in response to methylphenidate or amphetamine challenge with the reduction of dopamine release inversely correlating with the severity of cannabis dependence (Volkow et al. 2014) and cognitive deficits such as working memory impairments (Van De Giessen et al. 2017). Interestingly, a separate study reported no altered dopamine release that involved recently abstinent cannabis users with arguably less severe dependence (Urban et al. 2012). Further evidence has emerged of a positive correlation between stress-induced limbic striatal dopamine release and duration of cannabis use (Mizrahi et al. 2013). In another study, following a methylphenidate challenge, cannabis users displayed an attenuated striatal metabolic response with an inverse relationship between methylphenidate-induced metabolic increases and level of cannabis dependence (Wiers et al. 2016). Finally, evidence has also emerged of reductions in dopamine transporter (DAT) density in chronic cannabis users (Leroy et al. 2012).

Collectively, it would appear that the evidence derived from dopamine-based studies of chronic cannabis users complements the evidence of acute THC challenges. For example, where chronic cannabis use has been associated with reduced striatal dopamine synthesis capacity (Bloomfield et al. 2014), acute THC challenges report a significant reduction of radiotracer binding in the limbic striatum, consistent with increased dopamine levels in this region following relative to placebo. Combined, these results support the notion that increased striatal dopamine levels may be a mechanism underlying psychotic symptoms induced by THC. The weight of evidence in support of this idea is furthered from fMRI findings who report an association between the psychotomimetic effects of THC and the functional activation of striatal brain regions, commonly implicated in psychosis (Bhattacharyya et al. 2009; Bhattacharyya et al. 2012b; Bhattacharyya et al. 2012a).

## Summary and conclusions

The cannabinoid hypothesis of schizophrenia was proposed in 1996 (Emrich et al. 1996). It is premature to state that impaired reward processing underlies the relationship between cannabis use and onset and/or relapse of psychotic disorders such as schizophrenia. However, there is indication that reward pathways may have a role in the neuropathology of psychosis, which are influenced exogenous cannabinoids. Thus, based on evidence summarised above, specific investigation into this hypothesis is required.

Further to this, evidence has emerged of alterations in components of the endocannabinoid system in psychosis, particularly in terms of levels of CB1 receptors in key brain regions implicated in psychosis, as well as their circulating ligands. Some evidence also suggests that effect on the endocannabinoid system may underlie the effects of CBD, a cannabinoid that may have potential as an antipsychotic.

Aberrant salience (Kapur 2003), one of the prevailing theories of psychosis, suggests that psychotic symptoms arise from increases in spontaneous dopaminergic firing in mesolimbic reward pathways leading to abnormal stimulus-reinforcement (Miller 1976). This leads to alterations in the processing of rewarding stimuli resulting in the inappropriate assignment of motivational salience to contextually irrelevant stimuli (Kapur 2003). Consistent with this hypothesis, there is evidence of increased striatal presynaptic dopamine synthesis and release in psychosis, as well as growing neuroimaging evidence of abnormal engagement during reward processing of the striatum, a brain region rich in dopaminergic inputs, in psychosis. Although evidence from PET studies indicate only a modest effect of acute THC challenge on striatal dopamine, studies of cannabis users generally indicate impaired presynaptic dopaminergic function. Furthermore, a number of fMRI studies using reward processing and non-reward processing paradigms indicate that a single dose of THC may modulate key regions involved in reward and salience processing such as striatum, midbrain, insular, and anterior cingulate, with some of these effects correlating with the severity of THC-induced psychotic symptoms under experimental conditions. Complementing this, there is evidence that CBD may modulate some of the brain regions involved in reward/salience processing in an opposite direction to the effects of THC.

While the evidence summarised here may indicate a relationship between the neurobiology of psychotic disorders and the effects of cannabinoids on key neurocognitive and neurochemical substrates involved in the processing of rewarding stimuli, it remains to be seen whether this overlap is relevant and causally significant, or merely due to spurious similarities. Whether the modulation of reward processing and its neural substrates by THC may underlie its acute psychotomimetic effects remain unclear. Similarly, it remains to be seen whether any effects of CBD on reducing the severity of psychotic symptoms are related to its effects on reward processing and its neural substrates. Hence, future research should focus on addressing some of these unanswered questions using robust and well-powered experimental designs to understand the extent to which cannabinoid modulation of reward processing may underlie the symptoms of psychosis. This will also elucidate more generally the relationship that exists between endocannabinoid dysfunction, reward processing abnormalities, and psychosis.
